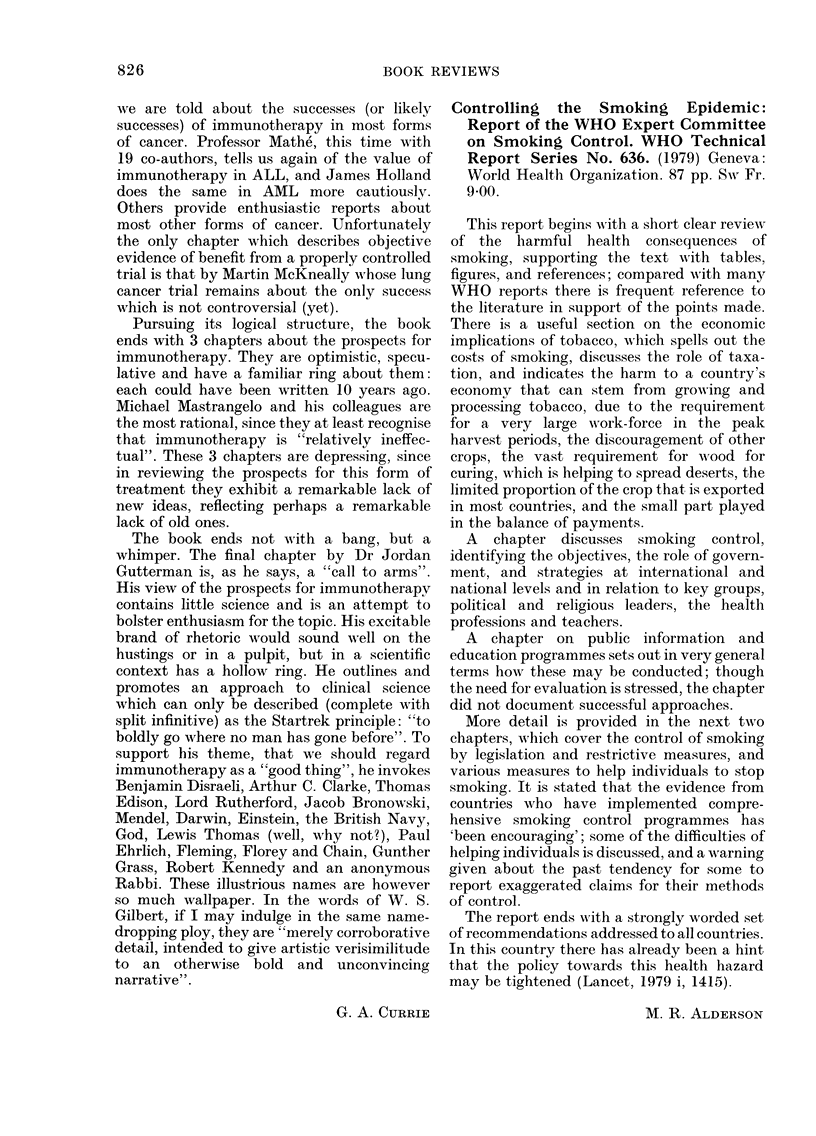# Controlling the Smoking Epidemic: Report of the WHO Expert Committee on Smoking Control. WHO Technical Report Series No. 636

**Published:** 1979-11

**Authors:** M. R. Alderson


**Controlling the Smoking Epidemic: Report of the WHO Expert Committee on Smoking Control. WHO Technical Report Series No. 636.** (1979) Geneva: World Health Organization. 87 pp. Sw Fr. 9·00.

This report begins with a short clear review of the harmful health consequences of smoking, supporting the text with tables, figures, and references; compared with many WHO reports there is frequent reference to the literature in support of the points made. There is a useful section on the economic implications of tobacco, which spells out the costs of smoking, discusses the role of taxation, and indicates the harm to a country's economy that can stem from growing and processing tobacco, due to the requirement for a very large work-force in the peak harvest periods, the discouragement of other crops, the vast requirement for wood for curing, which is helping to spread deserts, the limited proportion of the crop that is exported in most countries, and the small part played in the balance of payments.

A chapter discusses smoking control, identifying the objectives, the role of government, and strategies at international and national levels and in relation to key groups, political and religious leaders, the health professions and teachers.

A chapter on public information and education programmes sets out in very general terms how these may be conducted; though the need for evaluation is stressed, the chapter did not document successful approaches.

More detail is provided in the next two chapters, which cover the control of smoking by legislation and restrictive measures, and various measures to help individuals to stop smoking. It is stated that the evidence from countries who have implemented comprehensive smoking control programmes has 'been encouraging'; some of the difficulties of helping individuals is discussed, and a warning given about the past tendency for some to report exaggerated claims for their methods of control.

The report ends with a strongly worded set of recommendations addressed to all countries. In this country there has already been a hint that the policy towards this health hazard may be tightened (Lancet, 1979 i, 1415).

M. R. Alderson